# Data Mining in the U.S. National Toxicology Program (NTP) Database Reveals a Potential Bias Regarding Liver Tumors in Rodents Irrespective of the Test Agent

**DOI:** 10.1371/journal.pone.0116488

**Published:** 2015-02-06

**Authors:** Matthias Ring, Bjoern M. Eskofier

**Affiliations:** Digital Sports Group, Pattern Recognition Lab, Friedrich-Alexander-Universität, Erlangen-Nürnberg (FAU), Germany; National Chiao Tung University, TAIWAN

## Abstract

Long-term studies in rodents are the benchmark method to assess carcinogenicity of single substances, mixtures, and multi-compounds. In such a study, mice and rats are exposed to a test agent at different dose levels for a period of two years and the incidence of neoplastic lesions is observed. However, this two-year study is also expensive, time-consuming, and burdensome to the experimental animals. Consequently, various alternatives have been proposed in the literature to assess carcinogenicity on basis of short-term studies. In this paper, we investigated if effects on the rodents’ liver weight in short-term studies can be exploited to predict the incidence of liver tumors in long-term studies. A set of 138 paired short- and long-term studies was compiled from the database of the U.S. National Toxicology Program (NTP), more precisely, from (long-term) two-year carcinogenicity studies and their preceding (short-term) dose finding studies. In this set, data mining methods revealed patterns that can predict the incidence of liver tumors with accuracies of over 80%. However, the results simultaneously indicated a potential bias regarding liver tumors in two-year NTP studies. The incidence of liver tumors does not only depend on the test agent but also on other confounding factors in the study design, e.g., species, sex, type of substance. We recommend considering this bias if the hazard or risk of a test agent is assessed on basis of a NTP carcinogenicity study.

## Introduction

The U.S. National Toxicology Program (NTP) conducts carcinogenicity studies in rodents to identify substances that may be hazardous to humans [1–3]. In a typical carcinogenicity study, mice and rats of both sexes are exposed to a substance of interest. The substance is administered to the rodents at three dose levels for a period of two years. The three dose levels are defined on basis of preceding dose finding studies, and 50 rodents of every species and every sex are usually exposed to every dose level. The same amount of rodents is observed as controls which are not exposed to the substance.

This type of carcinogenicity study (CS) is currently the benchmark method to assess carcinogenicity [4]. It is motivated by the fact that all human carcinogens have also produced positive results in at least one animal model [5]. However, a two-year carcinogenicity study (2Y-CS) is also a high-cost and time-consuming procedure. Consequently, various alternative approaches have been discussed to identify carcinogenic substances [6, 7].

For example, quantitative structure-activity relationship (QSAR) models examine chemical properties of substances to predict the carcinogenic potential [8–15]. Another approach exploits findings from short-term CSs, from the beginning of 2Y-CSs, or from the preceding dose finding studies to predict the outcome of the 2Y-CS. For example, logistic regression was used to predict tumors in control animals based on their body weight at the beginning of a 2Y-CS [16, 17]. For male rats, the incidence of exacerbated chronic progressive nephropathy in dose finding studies was used to predict renal tubule tumors in 2Y-CSs [18]. For rats of both sexes, histopathological findings in 6- and 12-month CSs were used to predict carcinogenicity in 2Y-CSs [19]. For mice and rats of both sexes, hepatocellular lesions and increased liver weight in dose finding studies were combined to predict liver carcinogenicity in 2Y-CSs [20]. A later study also confirmed this approach and extended it to lung and kidney tumors [21].

In the present analysis, data mining methods were employed to predict liver tumors in 2Y-CSs using findings from the preceding dose finding studies. The focus on liver tumors was motivated from three perspectives. From an anatomical perspective, the liver is the organ where orally administered substances are transported to after absorption through the small intestine [22]. From a physiological perspective, the liver is the organ that is responsible for detoxification [23]. From a statistical perspective, the liver is the organ with the most positive carcinogenic results if all NTP studies are summarized [24].

In detail, the present analysis considered 138 NTP studies including mice and rats of both sexes. In addition to previous studies on the prediction of liver carcinogenicity [20, 21], the influence of different dose levels was also considered. In contrast to previous studies [20, 21], liver tumors were predicted on the finer data level of individual animals instead of summary statistics for entire 2Y-CSs.

The results revealed patterns that can predict the incidence of liver tumors in 2Y-CSs on basis of findings from dose finding studies, i.e., findings from short-term CSs. However, these patterns simultaneously indicated a potential bias regarding liver carcinogenicity in the 2Y-CS. The patterns illustrated that the incidence of liver tumors in 2Y-CS does not only depend on the test substance, i.e., the subject of investigation. For instance, the results indicated that male mice, which are exposed to single substances at a high dose level, will likely develop a liver tumor. In contrast, the results did not indicate the same tendency for female mice, which are exposed to single substances at a high dose level. Thus, an increase in liver tumors in male mice may not be as alerting as a comparative increase in female mice if both sexes are exposed to identical substances at identical high dose levels. This bias should be considered in the statistical evaluation of a 2Y-CS because the decision on carcinogenicity of a substance is based on this evaluation.

## Methods

### Data Set

The NTP provides data from CSs in two forms. The technical report (TR, [25]) for every CS includes a statistical analysis and the decision on carcinogenicity of the test substance. The NTP database (CarTox, [26]) includes detailed data for every animal used in every CS. At the time of the present analysis, TRs for 582 CSs were available. Based on these TRs and the CarTox database, a data set was built using the following three steps.

**Filtering of Technical Reports**. The TRs for all 582 CSs were evaluated for inclusion in the present analysis. Four inclusion criteria were specified on every CS. First, every CS was required to be labeled as a long-term CS, which usually indicates a duration of 104 weeks. Second, every CS was required to administer the substance on an oral route, which was either dosed-feed, dosed-water, gavage, or micro-encapsulation in feed. An oral route was necessary so that the substance was directly transported to the target organ, the liver, after administration. Third, every CS was required to be preceded by a dose finding study, which was usually conducted for a period of 13 weeks. Fourth, the TR for every CS was required to provide liver weight recordings from the dose finding study. The third and fourth criteria were necessary because these liver weight recordings were employed as prediction attributes (see below).

Some TRs provided liver weight recordings only for some animal groups ( e.g., TR No. 373 provided data for rats but not for mice). In such cases, the CS was included but animal groups with missing data were removed later (see below).

The inclusion criteria yielded a subset of 138 2Y-CSs (Tables 1, 2, 3). Two more 2Y-CSs (TR No. 278, TR No. 244) also fulfilled all inclusion criteria. However, they were not included because the corresponding data was not found in the CarTox database.

**Table 1:** List of 2Y-CSs on single substances that were included in the analysis.

**TR**	**CASRN**	**Substance**
TR-243	79-01-6	Trichloroethylene
TR-298	597-25-1	Dimethyl morpholinophosphoramidate
TR-308	108171-26-2	Chlorinated paraffins: C12, 60% chlorine
TR-320	83-79-4	Rotenone
TR-325	82-68-8	Pentachloronitrobenzene
TR-328	598-55-0	Methyl carbamate
TR-332	149-30-4	2-Mercaptobenzothiazole
TR-333	135-88-6	N-Phenyl-2-naphthylamine
TR-334	121-88-0	2-Amino-5-nitrophenol
TR-337	59-87-0	Nitrofurazone
TR-339	99-57-0	2-Amino-4-nitrophenol
TR-341	67-20-9	Nitrofurantoin
TR-345	121-19-7	Roxarsone
TR-348	41372-08-1	Methyldopa sesquihydrate
TR-352	924-42-5	N-Methylolacrylamide
TR-354	828-00-2	Dimethoxane
TR-356	54-31-9	Furosemide
TR-357	58-93-5	Hydrochlorothiazide
TR-358	303-47-9	Ochratoxin A
TR-359	298-81-7	8-Methoxypsoralen
TR-361	67-72-1	Hexachloroethane
TR-365	78-11-5	Pentaerythritol tetranitrate
TR-366	123-31-9	Hydroquinone
TR-367	50-33-9	Phenylbutazone
TR-368	389-08-2	Nalidixic acid
TR-369	98-85-1	alpha-Methylbenzyl alcohol
TR-372	20325-40-0	3,3’-Dimethoxybenzidine dihydrochloride
TR-373	108-30-5	Succinic anhydride
TR-381	2244-16-8	D-Carvone
TR-383	81-49-2	1-Amino-2,4-dibromoanthraquinone
TR-384	96-18-4	1,2,3-Trichloropropane
TR-387	60-13-9	DL-amphetamine sulfate
TR-389	26628-22-8	Sodium azide
TR-391	115-96-8	tris(2-Chloroethyl) phosphate
TR-392	CHLORAMINEMX	Chloraminated water
TR-393	7681-49-4	Sodium fluoride
TR-394	103-90-2	Acetaminophen (4-hydroxyacetanilide)
TR-395	57-66-9	Probenecid
TR-396	79-11-8	Monochloroacetic acid
TR-399	1271-19-8	Titanocene dichloride
TR-401	137-09-7	2,4-Diaminophenol dihydrochloride
TR-402	110-00-9	Furan
TR-403	108-46-3	Resorcinol
TR-404	57-41-0	5,5-Diphenylhydantoin (phenytoin)
TR-405	6459-94-5	C.I. Acid red 114
TR-406	96-48-0	gamma-Butyrolactone
TR-407	2425-85-6	C.I. Pigment red 3
TR-407	2429-74-5	C.I. Direct blue 15
TR-408	7487-94-7	Mercuric chloride
TR-409	117-39-5	Quercetin
TR-411	6471-49-4	C.I. Pigment red 23
TR-412	7336-20-1	4,4’-Diamino-2,2’-stilbenedisulfonic acid, disodium salt
TR-413	107-21-1	Ethylene glycol
TR-414	1825-21-4	Pentachloroanisole
TR-415	9005-65-6	Polysorbate 80 (glycol)
TR-416	91-23-6	o-Nitroanisole
TR-418	100-01-6	p-Nitroaniline
TR-419	59820-43-8	HC yellow 4
TR-420	396-01-0	Triamterene
TR-422	91-64-5	Coumarin
TR-423	119-84-6	3,4-Dihydrocoumarin
TR-424	120-32-1	o-Benzyl-p-chlorophenol
TR-425	58-33-3	Promethazine hydrochloride
TR-428	10034-96-5	Manganese sulfate monohydrate
TR-430	28407-37-6	C.I. Direct blue 218
TR-431	140-11-4	Benzyl acetate
TR-432	10326-27-9	Barium chloride dihydrate
TR-433	1330-78-5	Tricresyl phosphate
TR-435	96-69-5	4,4-Thiobis(6-tert-butyl-m-cresol)
TR-436	75-65-0	tert-Butyl alcohol
TR-439	298-59-9	Methylphenidate hydrochloride
TR-442	62-23-7	p-Nitrobenzoic acid
TR-443	604-75-1	Oxazepam
TR-445	6533-68-2	Scopolamine hydrobromide trihydrate
TR-446	1972-08-3	1-trans-delta-9-Tetrahydrocannabinol
TR-452	3296-90-0	2,2-bis(Bromomethyl)-1,3-propanediol
TR-455	76-57-3	Codeine
TR-457	599-79-1	Salicylazosulfapyridine
TR-458	85-68-7	Butyl benzyl phthalate
TR-459	1948-33-0	t-Butylhydroquinone
TR-463	8003-22-3	D & C yellow no. 11
TR-465	77-09-8	Phenolphthalein
TR-468	604-75-1	Oxazepam
TR-469	30516-87-1	3’-Azido-3’-deoxythymidine (AIDS)
TR-470	110-86-1	Pyridine
TR-473	58-55-9	Theophylline
TR-476	125-33-7	Primidone (primaclone)
TR-477	127-00-4	1-Chloro-2-propanol, technical
TR-483	87-86-5	Pentachlorophenol, purified

**TR**	**CASRN**	**Substance**
TR-485	434-07-1	Oxymetholone
TR-491	93-15-2	Methyleugenol
TR-493	518-82-1	Emodin
TR-494	84-65-1	Anthraquinone
TR-495	7632-00-0	Sodium nitrite
TR-496	116355-83-0	Fumonisin B1
TR-501	80-07-9	p,p’-Dichlorodiphenyl sulfone
TR-505	5392-40-5	Citral
TR-506	107-13-1	Acrylonitrile
TR-509	142-83-6	2,4-Hexadienal
TR-511	25265-71-8	Dipropylene glycol
TR-512	37319-17-8	Elmiron (sodium pentosanpolysulfate)
TR-516	693-98-1	2-Methylimidazole
TR-517	7775-09-9	Water disinfection byproducts (Sodium chlorate)
TR-520	57465-28-8	Toxic equivalency factor evaluation ((PCB 126) 3,3’,4,4’,5-pentachlorobiphenyl)
TR-521	1746-01-6	TEF evaluation (TCDD)
TR-525	57117-31-4	TEF evaluation (PECDF (Pentachlorodibenzofuran))
TR-529	35065-27-1	Toxic equivalency factor evaluation (PCB 153- 2,2’-4,4’,5,5’-hexachlorobiphenyl)
TR-532	75-27-4	Water disinfection byproducts (Bromodichloromethane)
TR-537	631-64-1	Water disinfection byproducts (Dibromoacetic acid)
TR-539	446-72-0	Endocrine disruptor (Genistein)
TR-540	7220-79-3	Methylene blue trihydrate
TR-541	75-12-7	Formamide
TR-544	3252-43-5	Water disinfection byproducts (Dibromoacetonitrile)
TR-549	5589-96-8	Water disinfection byproducts (Bromochloroacetic acid)
TR-551	97-54-1	Isoeugenol
TR-554	67-47-0	5-(Hydroxymethyl)-2-furfural
TR-556	27882-76-4	Chromium picolinate monohydrate
TR-557	123-35-3	beta-Myrcene
TR-558	14047-09-7	3,3’,4,4’-Tetrachloroazobenzene
TR-559	31508-00-6	TEF evaluation (PCB 118)
TR-560	63-05-8	Androstenedione
TR-563	89-82-7	Pulegone
TR-570	76231-76-0	alpha/beta Thujone mixture
TR-573	SANTRIMER2	Styrene-acrylonitrile trimer
TR-575	79-06-1	Acrylamide
TR-579	99-97-8	N,N-Dimethyl-p-toluidine
TR-580	108-99-6	beta-Picoline

The left column shows the name of all Technical Reports (TR) that were included in the analysis. The middle column shows the Chemical Abstracts Service Registry Number (CASRN) of the test substance, as termed in the CarTox database. The right column shows the name of the test substance, as termed in the CarTox database.

**Table 2:** List of 2Y-CSs on mixtures that were included in the analysis.

**TR**	**CASRN**	**Substance**
TR-305	108171-27-3	Chlorinated paraffins: C23, 43% chlorine
TR-398	67774-32-7	Polybrominated biphenyl mixture (Firemaster FF-1)
TR-526	TEFDIOXINMIX	TEF evaluation (Dioxin mixture)
TR-531	TEFPCBMIX	TEF Evaluation (PCB Mixture; PCB 126/PCB 118)

The left column shows the name of all Technical Reports (TR) that were included in the analysis. The middle column shows the Chemical Abstracts Service Registry Number (CASRN) of the test substance, as termed in the CarTox database. The right column shows the name of the test substance, as termed in the CarTox database.

**Table 3:** List of 2Y-CSs on multi-compounds that were included in the analysis.

**TR**	**CASRN**	**Substance**
TR-426	538-23-8	Tricaprylin
TR-426	8001-23-8	Safflower oil
TR-427	8024-37-1	Turmeric, oleoresin (curcumin)
TR-562	GOLDENSEALRT	Goldenseal root powder
TR-565	84604-20-6	Milk thistle extract
TR-567	50647-08-0	Ginseng
TR-571	9000-38-8	Kava kava extract
TR-577	ALOEVLEAFEXT	Aloe vera whole leaf extract (native)
TR-578	90045-36-6	Ginkgo biloba extract

The left column shows the name of all Technical Reports (TR) that were included in the analysis. The middle column shows the Chemical Abstracts Service Registry Number (CASRN) of the test substance, as termed in the CarTox database. The right column shows the name of the test substance, as termed in the CarTox database.

**Combination of Technical Reports and Animal Data**. For every animal used in any of the 138 2Y-CSs, five attributes were extracted from the CarTox database. First, the species (SP) of the animal (mouse, rat). Second, the sex (SE) of the animal (female, male). Third, the indicator for control (CO) animals (true, false). Fourth, the removal reason (RR) of the animal from the 2Y-CS ( e.g., terminal sacrifice, natural death). Fifth, the incidence of at least one primary liver tumor (LT) in the histopathological examination (true, false). Table 4 specifies our distinction between primary and non-primary liver tumors.

**Table 4:** Distinction between primary and non-primary liver tumors.

**Diagnosis**	**Liver**	**Diagnosis**	**Liver**
Acinar-Cell Carcinoma, Metastatic	yes	Leukemia Mononuclear	no
Adenocarcinoma	yes	Leukemia Myeloid	no
Adenocarcinoma, Nos	yes	Leukemia, Mononuclear Cell	no
Adenocarcinoma, Nos, Metastatic	no	Lipoma	yes
Adenoma	yes	Liposarcoma	yes
Adenoma, Nos	yes	Lymphoma Malignant	no
Alveolar/Bronchiolar Carcinoma	no	Lymphoma Malignant Histiocytic	no
Bile Duct Carcinoma	yes	Lymphoma Malignant Lymphocytic	no
Carcinoid Tumor Malignant	yes	Lymphoma Malignant Mixed	no
Carcinoma	yes	Lymphoma Malignant Undifferentiated Cell Type	no
Carcinoma, Nos, Metastatic	no	Lymphoma, Histiocytic-Malignant Type	no
Chemodectoma Malignant	no	Lymphoma, Lymphocytic-Malignant Type	no
Cholangiocarcinoma	yes	Lymphoma, Mixed-Malignant Type	no
Cholangioma	yes	Lymphoma, Nos-Malignant	no
Choriocarcinoma	no	Lymphoma, Undifferentiated-Malignant Type	no
Endometrial Stromal Sarcoma, Metastatic	no	Mast Cell Tumor Malignant	no
Fibrosarcoma	yes	Mast Cell Tumor Nos	no
Fibrosarcoma, Metastatic	no	Mesothelioma Malignant	yes
Fibrous Histiocytoma	no	Mesothelioma NOS	yes
Fibrous Histiocytoma, Metastatic	no	Mixed Hepato/Cholangio Carcinoma	yes
Granulosa Cell Tumor Malignant	no	Mixed Tumor Malignant	yes
Hemangioma	yes	Myxoma	no
Hemangiosarcoma	yes	Neoplasm NOS	yes
Hemangiosarcoma, Metastatic	no	Neoplastic Nodule	yes
Hemangiosarcoma, Uncertain Primary Or Metastatic	yes	Neurilemoma, Metastatic	no
Hepatoblastoma	yes	Neuroblastoma	no
Hepatocellular Adenoma	yes	Neuroendocrine Tumor, Malignant	no
Hepatocellular Carcinoma	yes	Neurofibrosarcoma, Metastatic	no
Hepatocholangiocarcinoma	yes	Osteosarcoma	no
Hepatocholangioma	yes	Osteosarcoma, Metastatic	no
Histiocytic Sarcoma	no	Pheochromocytoma Malignant	no
Islet-Cell Carcinoma, Metastatic	no	Plasma Cell Tumor Malignant	yes
Ito Cell Tumor Benign	yes	Rhabdomyosarcoma	no
Ito Cell Tumor Malignant	yes	Sarcoma	yes
Ito Cell Tumor Nos	yes	Sarcoma Stromal	no
Kupffer-Cell Sarcoma	yes	Sarcoma, Nos	yes
Leiomyosarcoma	no	Sarcoma, Nos, Metastatic	no
Leukemia	no	Sarcoma, Nos, Uncertain Primary Or Meta	yes
Leukemia Erythrocytic	no	Schwannoma Malignant	yes
Leukemia Granulocytic	no	Squamous Cell Carcinoma	no
Leukemia Lymphocytic	no	Thymoma Malignant	no
Leukemia Megakaryocytic	no	Yolk Sac Carcinoma	Yes
Leukemia Monocytic	No		

The left column shows all different neoplastic diagnoses, as termed in the CarTox database. The right column shows the distinction between primary and non-primary liver tumors (which was provided by toxicological experts).

The five database attributes were combined with two attributes from the TRs. First, the information if the administered substance (SU) was a single substance, a mixture, or a multi-compound (single, mixture, multi-compound). This information was included because carcinogenicity of multi-compounds, which were mostly herbal medicines, is currently discussed in the literature [27, 28]. Second, the information if the administered dose level (DL) indicated potential liver toxicity in the dose finding study (toxic, non-toxic, missing data). Potential liver toxicity for a dose level was declared if the TR reported a statistically significant increase in liver weight in the dosing finding study for this dose level, or the nearest lower dose level, in the group of animals that was exposed to this dose level compared to the group of control animals. The liver weight was utilized for this purpose because increases in liver weight were observed to be associated with hepatomegaly, increased enzyme induction, and increased mitogenesis [29–31]. These three factors were, in turn, reported as potential early indicators of (non-genotoxic) liver tumors [32]. Thus, increased liver weight may indicate potential liver toxicity and, consequently, the development of liver tumors.

This representation for the dose level using only two categories (toxic and non-toxic) provided a consistent descriptor across all 2Y-CSs, in contrast to the specification of the dose level in form of a concrete number. This is because of two reasons. First, carcinogenic activity of two different substances may not be identical, even if the substances are administered at identical dose levels. Second, the dose level was reported in a variety of different notations and units ( e.g., about 600 different specifications for the dose level were found in the CarTox database for the considered 2Y-CSs).

This procedure yielded a data set of 116673 (test substance exposed and control) animals, and every animal was described by seven attributes (SP, SE, CO, RR, LT, SU, DL). There are more attributes available in the CarTox database to describe animals. However, these attributes were either not suitable for the present analysis, e.g., the Chemical Abstracts Service Registry Number (CASRN), or their attribute values were correlated with certain TRs, e.g., the strain, because some strains were employed only in specific TRs.

**Filtering of Animal Data**. All 116673 animals were evaluated for inclusion in the analysis. Three inclusion criteria were specified on every animal. First, every animal was required to be exposed to the test substance, i.e., the attribute CO was required to be false. Second, the dose level for every animal was required to be available in the above mentioned representation, i.e., animals with missing data in the attribute DL were excluded. Third, every animal was required to be removed from the 2Y-CS because of an ordinary reason (attribute RR). For example, animals that were removed from a 2Y-CS because they drowned were excluded. Table 5 lists all removal reasons and specifies our distinction between ordinary and non-ordinary reasons.

**Table 5:** Reasons for removal of an animal from a 2Y-CS.

**Removal reason**	**Inclusion in this analysis**
Aborted	no
Accident	no
Accidently Killed	no
Dead	yes
Dosing Accident	no
Drowned	no
Gavage Death	no
Harvest	no
Interval Sacrifice	yes
Mis-Sexed	no
Missing	no
Moribund	yes
Moribund Sacrifice	yes
Natural Death	yes
Other	no
Scheduled Sacrifice	yes
Special Control	no
Special Study	no
Surplus	no
Terminal Sacrifice	yes
Wrong Sex	no

The left column shows all different reasons for the removal of an animal from a 2Y-CS, as termed in the CarTox database. The right column shows which animals were included in the analysis. (The distinction was provided by toxicological experts.)

The inclusion criteria yielded a subset of 68778 (test substance exposed) animals, and every animal was described by five attributes (SP, SE, LT, SU, DL). The attributes CO and RR were removed because they were not used again after the filtering procedure.

### Data Mining

Data mining methods were employed to predict for every animal if at least one primary liver tumor (LT) will be diagnosed at the end of the 2Y-CS. The predictions were performed using information about the animal (SE, SP), specifications on the 2Y-CS (SU), and findings from the dose finding study (DL). No findings from the 2Y-CS itself were employed to perform predictions.

If this approach results in a positive outcome, it may not only indicate that the incidence of liver tumors can be predicted on basis of short-term CSs. It might also indicate a potential bias regarding liver carcinogenicity in the 2Y-CS. This is because the predictions are independent of the test article, i.e., the actual subject of investigation. The predictions are only dependent on factors (SE, SP, SU, DL) that are specified and controlled by the conductors of the 2Y-CSs.

**Algorithms**. The C4.5 algorithm [33] was employed as the primary data mining algorithm. It creates a decision tree to predict the incidence of liver tumors and was selected because decision trees provide a simple and reasonable basis for further mechanistic interpretations [34]. There are also other algorithms that create decision trees, however, the C4.5 algorithm is one of the most popular algorithms [35, Ch. 8.4.2].

**Figure 1:**
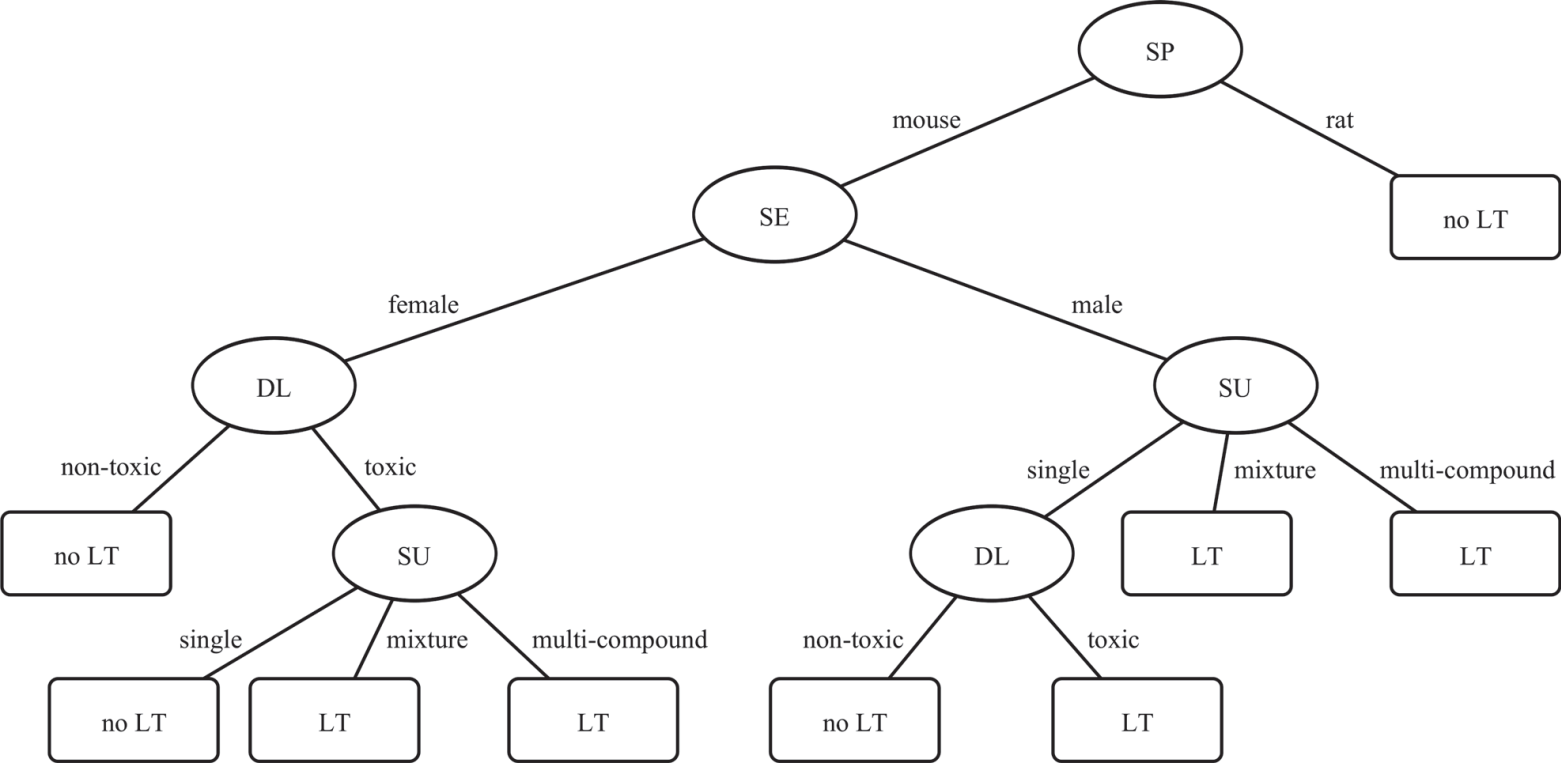
Decision tree to predict liver tumors. The tree was learned using the C4.5 algorithm. It predicts liver tumors (LT) with information about the animal (SP = species, SE = sex), specifications on the 2Y-CS (SU = substance), and an indicator for liver toxicity extracted from the dose finding study (DL = dose level).

In brief, a decision tree predicts the incidence of liver tumors for every animal by querying its input attributes (DL, SE, SP, SU) in a tree-formed manner (for an illustration, see Fig. 1 in the results section). Every inner node (oval-shaped nodes in Fig. 1) represents a query on an attribute. Every leaf (rectangular-shaped nodes in Fig. 1) represents a prediction. Thus, the prediction for an animal (LT, no LT) is given by the leaf that is reached at the end of the animal’s path through the tree.

Roughly speaking, the C4.5 algorithm uses the following method to create a decision tree for a given set of animals. Every query on an input attribute will split the set of animals into two, or more, subsets. For every attribute and the corresponding subsets, the algorithm computes a measure of impurity (gain ratio, see, e.g., [36, Ch. 4.3]). This measures becomes minimal if the animals in every subset have the same target value, and maximal if the amount of animals with liver tumors and the amount of animals without liver tumors is identical. Beginning with the entire set of animals, the algorithm declares the attribute as the upmost tree node that minimizes the impurity. Then, the algorithm recursively processes every branch of the upmost node in the same fashion.

The C4.5 decision tree will form the basis for mechanistic interpretations in the following sections. In addition, further algorithms were employed to examine stability of predictions on the present data set. These algorithms were AdaBoost [37], PART [38], and Random Forests [39]. In brief, AdaBoost combines several “weak” algorithms to build a “strong” algorithm. In the present analysis, the weak algorithms were decision stumps, i.e., decision trees with exactly one node, and the number of weak algorithms was set to ten. In the following, the abbreviation AdaBoost-DS refers to the AdaBoost algorithm utilizing decision stumps. PART generates several “partial” decision trees and extracts decision rules from these partial trees. Random Forests create several “random” decision trees and output the majority decision of all individual trees. In the present analysis, the number of random trees was set to ten, and the number of available attributes for every split was set to three (according to the formula log_c_ *M* + 1 = 3, where *M* = 4 is the number of attributes, see [39]).

The WEKA software (version 3.6.8, [40]) was used to perform all algorithms.

**Settings**. Three settings were examined. First, all algorithms were applied to the set of all 68778 animals (SET1). This setting included 15029 animals with liver tumors and 53749 animals without liver tumors. Second, all algorithms were applied to the set of animals that was exposed to a multi-compound (SET2). This setting was examined because carcinogenicity of multi-compounds, which were mostly herbal medicines, is currently discussed in the literature [27, 28]. There were a total of 4532 animals exposed to multi-compounds, and these animals originated from 8 different studies (Table 3). This setting included 1129 animals with liver tumors and 3403 animals without liver tumors. Third, all algorithms were applied to the set of mice that was exposed to a multi-compound (SET3). This setting was examined because the decision tree for SET2 predicted liver tumors only for mice (see results section). There were a total of 2135 mice exposed to multi-compounds. This setting included 1046 animals with liver tumors and 1089 animals without liver tumors.

Both SET1 and SET2 include more animals without liver tumors than animals with liver tumors. In such situations, a subset of the larger class is often randomly sampled to have balanced distributions. However, in the present analysis, the imbalanced classes are explicitly employed to provide the information that liver tumors are less frequent to the algorithms.

**Prediction Performance**. Three performance measurements were computed for every setting and every algorithm. First, prediction accuracy was computed as the sum of true positives and true negatives divided by the sum of all positives and all negatives. Second, sensitivity was computed as true positives divided by all positives. Third, specificity was computed as true negatives divided by all negatives. Both sensitivity and specificity have the advantage that they are, per definitionem, not affected by imbalanced class distributions, which are present in SET1 and SET2. Furthermore, the absolute amount of true positives, true negatives, false positives, and false negatives were recorded in the form of confusion matrices.

The performance measurements were computed using a stratified 10-fold cross-validation [41, Ch. 7.10]. In brief, the cross-validation procedure simulates the application scenario for every algorithm. For this purpose, the data set is randomly partitioned into a training and a testing set. Every algorithm learns its prediction schema on the training set and tests it on the testing set. Then, the performance measurements are computed for the results on the testing set.

The 10-fold cross-validation repeats this procedure ten times to get averaged estimations for the performance measurements. For this purpose, the data set is partitioned into ten equally-sized subsets. Every subset is used once for testing while the remaining nine subsets are used for training. Then, the means of the ten individual performance measurements give the averaged estimations for the performance measurements. To ensure identical conditions for all algorithms, ten identical (but randomly defined) subsets were employed for all algorithms.

The stratified 10-fold cross-validation creates the ten subsets such that the ratio between animals with liver tumors and animals without liver tumors is preserved with respect to the entire data set.

The WEKA software (version 3.6.8, [40]) was used to perform the stratified 10-fold cross-validation.

**Statistical Significance of Predictions**. The performance measures in the previous section describe the algorithms’ ability to predict the incidence of liver tumors. If the performance measures are adequately high, these algorithms could be employed in practical applications. If the performance measures are not adequately high, the results may still provide insights into the data set. As long as the predictions are significantly better than trivial heuristics, the algorithms’ prediction schemas represent insights between the input attributes (DL, SE, SP, SU) and the target attribute (LT) that were extracted from the data set.

Therefore, predictions with the C4.5 decision tree were deemed significant if they were significantly better than trivial heuristics, i.e., random guessing and majority voting. Random guessing means that a liver tumor is predicted with probability *s_1_* = 0.5, regardless of the attribute values of an animal. Majority voting means that the same target value is constantly predicted, regardless of the attribute values of an animal. This target value is the one that most frequently occurred in the training set.

To compare predictions by the decision tree to these trivial heuristics, a binomial test was applied, because predictions with the decision tree can be modeled by a Bernoulli process [36, Ch. 5.2]. In terms of a Bernoulli process, true positives and true negatives represent successes, while false positives and false negatives represent failures. The probability of a drawing a success from a Bernoulli process is called success probability π. In this setting, the null hypothesis of a binomial test states that the empirically observed success probability, i.e., the prediction accuracy of the decision tree, is identical to the true success probability π, i.e., the success probability of random guessing or majority voting.

Therefore, the null hypothesis that predictions with the decision tree are identical to random guessing was tested by setting the success probability π to *s_1_* = 0.5. The null hypothesis that predictions with the decision tree are identical to majority voting was tested by setting the success probability π to *s_2_*. This probability *s_2_* was estimated by using an algorithm that actually performed majority voting. The accuracy of this algorithm was estimated with a stratified 10-fold cross-validation, and the success probability *s_2_* was set to the prediction accuracy. The ten subsets in this cross-validation were identical to the subsets of the other algorithms (see previous section).

For both binomial tests, the empirical number of successes was set to the sum of true positives and true negatives from the cross-validation procedure for the C4.5 algorithm. The significance level was set to *p*<0.01.

The R software (version 3.0.2, [42]) was used to perform the statistical tests. The WEKA software (version 3.6.8, [40]) was used to perform the majority voting algorithm.

## Results

For the C4.5 algorithm applied to SET1, the cross-validation estimated an accuracy of 80.6%, sensitivity of 27.8%, and specificity of 95.4% (Table 6; confusion matrix in Table 7). The decision trees in all ten folds of the cross-validation procedure were identical (Fig. 1). These trees may be interpreted as follows: For rats, the decision tree predicted no liver tumors at all. For mice, the tree first differentiated between females and males. For female mice, the tree predicted liver tumors if a mixture or a multi-compound was administered at a dose level which indicated liver toxicity in the dose finding study. For male mice, the tree predicted liver tumors if a mixture or a multi-compound was administered, or if the dose level indicated liver toxicity in the dose finding study.

**Table 6:** Performance measures for the C4.5 algorithm.

	**C4.5 decision tree**			**Significance compared to**	
	**accuracy (%)**	**sensitivity (%)**	**specificity (%)**	**random guessing**	**majority voting**
SET1	80.6 ± 0.2	27.8 ± 1.0	95.4 ± 0.0	*p<0.001*	*p<0.001*
SET2	82.7 ± 1.2	79.0 ± 4.2	84.0 ± 0.4	*p<0.001*	*p<0.001*
SET3	67.3 ± 1.9	85.3 ± 3.5	50.0 ± 0.9	*p<0.001*	*p<0.001*

SET1 denotes the setting in which all animals were employed. SET2 denotes the setting in which only animals exposed to multi-compounds were employed. SET3 denotes the setting in which only mice exposed to multi-compounds were employed. The performance measurements were estimated using a stratified 10-fold cross-validation.

**Table 7:** Confusion matrices for the C4.5 algorithm.

**SET1**		**SET2**		**SET3**	
4171	10858	892	237	892	154
2468	51281	545	2858	545	544

SET1 denotes the setting in which all animals were employed. SET2 denotes the setting in which only animals exposed to multi-compounds were employed. SET3 denotes the setting in which only mice exposed to multi-compounds were employed. The confusion matrices were computed using a stratified 10-fold cross-validation. Every confusion matrix shows the number of true positives and false negatives in the first row, and the number of false positives and true negatives in the second row.

**Figure 2:**
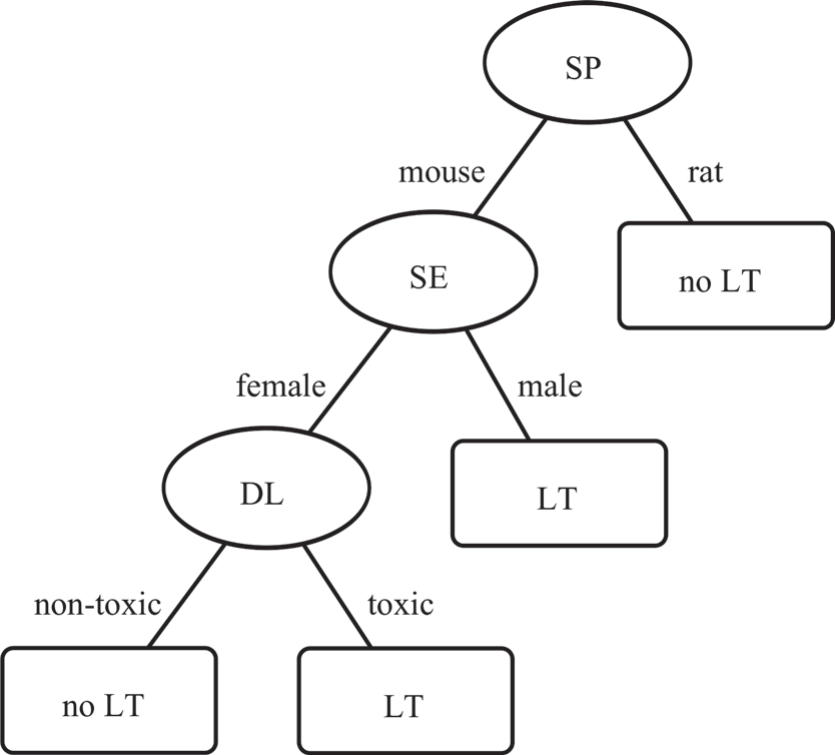
Decision tree to predict liver tumors in case of multi-compounds. The tree was learned using the C4.5 algorithm. It predicts liver tumors (LT) with information about the animal (SP = species, SE = sex), and an indicator for liver toxicity extracted from the dose finding study (DL = dose level).

For the C4.5 algorithm applied to SET2, the cross-validation estimated an accuracy of 82.7%, sensitivity of 79.0%, specificity of 84.0% (Table 6; confusion matrix in Table 7). The decision trees in all ten folds of the cross-validation procedure were identical (Fig. 2). These trees predicted liver tumors in the same situations as the decision trees for SET1 in case of animals exposed to multi-compounds.

For the C4.5 algorithm applied to SET3, the cross-validation estimated an accuracy of 67.3%, sensitivity of 85.3%, and specificity of 50.0% (Table 6; confusion matrix in Table 7). The decision trees in all ten folds of the cross-validation procedure were identical and also identical to the decision tree for SET2 in case of mice (Fig. 2).

For all three settings, predictions with the C4.5 algorithm were significantly better than trivial heuristics (Table 6).

For the PART and Random Forest algorithms, the performance measures were similar to the C4.5 algorithm, in case of all three settings (Table 8). For the AdaBoost-DS algorithm applied to SET1 and SET2, the sensitivity slightly decreased while the specificity slightly increased (Table 8). For the AdaBoost-DS algorithm applied to SET3, the performance measures were similar to all other algorithms (Table 8).

**Table 8:** Performance measures for the AdaBoost-DS, PART, and Random Forest algorithms.

	**AdaBoost-DS**		
	**accuracy (%)**	**sensitivity (%)**	**specificity (%)**
SET1	80.5 ± 0.2	23.9 ± 0.9	96.3 ± 0.0
SET2	82.3 ± 0.9	61.8 ± 2.9	89.0 ± 0.4
SET3	67.3 ± 1.9	85.3 ± 3.5	50.0 ± 0.9
	PART		
	accuracy (%)	sensitivity (%)	specificity (%)
SET1	80.6 ± 0.2	27.8 ± 1.0	95.4 ± 0.0
SET2	82.7 ± 1.2	79.0 ± 4.2	84.0 ± 0.4
SET3	67.3 ± 1.9	85.3 ± 3.5	50.0 ± 0.9
	Random Forest		
	accuracy (%)	sensitivity (%)	specificity (%)
SET1	80.6 ± 0.2	27.8 ± 1.0	95.4 ± 0.0
SET2	82.7 ± 1.2	79.0 ± 4.2	84.0 ± 0.4
SET3	67.3 ± 1.9	85.3 ± 3.5	50.0 ± 0.9

SET1 denotes the setting in which all animals were employed. SET2 denotes the setting in which only animals exposed to multi-compounds were employed. SET3 denotes the setting in which only mice exposed to multi-compounds were employed. The performance measurements were estimated using a stratified 10-fold cross-validation.

## Discussion

In the present analysis, data mining was employed to predict the incidence of liver tumors in 2Y-CSs. Several algorithms performed predictions with information about the animals, specifications on the 2Y-CS, and findings from the preceding dose finding study, but without findings from the 2Y-CS itself. Three settings were examined with this approach, and prediction accuracies of about 80%, 83%, and 67% were achieved in the three settings, respectively.

### Prediction Performance

For the C4.5 algorithm in SET1, the high specificity of 95% shows that most animals without liver tumors were recognized as such. A high specificity also indicates that the number of false positives was small. In other words, the C4.5 algorithm is very likely to be correct whenever it predicts a liver tumor, because only few animals without liver tumors were classified as animals with liver tumors. Hence, the following pattern may be extracted from the tree (Fig. 1): Female mice, which are exposed to mixtures or multi-compounds at a dose level that indicated liver toxicity, as well as male mice, which are exposed to mixtures or multi-compounds, or to single substances at a dose level that indicated liver toxicity, will very likely develop a liver tumor.

However, this pattern has to be interpreted in combination with the sensitivity. The low sensitivity of 27% shows that many animals with liver tumors were not recognized as such. This indicates that the above pattern will also miss many animals with liver tumors. In other words, there are more situations in which animals will develop liver tumors. An explanation for this low sensitivity might be that truly carcinogenic substances cause liver tumors regardless of the type of animal or dose level, i.e., regardless of the attributes available to the C4.5 algorithm. In fact, truly carcinogenic substances may cause liver tumors without necessarily increasing liver weight [43]. Thus, the C4.5 algorithm cannot detect such tumors on basis of the available attributes.

As the performance measures for the other algorithms (AdaBoost-DS, PART, Random Forests) were similar, it may be concluded that the present approach would miss many substances that cause liver tumors. Therefore, it cannot be recommended as an alternative to assess liver carcinogenicity in SET1. The general approach of SET1 was probably too optimistic for the high variety among 138 different TRs.

For the C4.5, PART, and Random Forest algorithms in SET2, both sensitivity and specificity were as promising as the prediction accuracy. Most animals with liver tumors were identified as such (80% sensitivity), and most animals without liver tumors were also identified as such (84% specificity).

Therefore, the relationship between liver tumors and multi-compounds was examined in more detail in SET3.

Because the C4.5 decision tree only predicted liver tumors in case of mice, only this species was further examined in SET3. The high sensitivity of 85% shows that most animals with liver tumors were recognized as such. A high sensitivity also indicates that the number of false negatives was small. In other words, the C4.5 algorithm is very likely to be correct whenever it predicts the absence of a liver tumor, because only few animals with liver tumors were classified as animals without liver tumors. Hence, the following pattern may be extracted from the decision tree (Fig. 2): Female mice, which are exposed to multi-compounds at a dose level that did not indicate liver toxicity, will very unlikely develop a liver tumor.

As in SET1, this pattern has to be interpreted in combination with the specificity. The rather low specificity of 50% shows that many animals without liver tumors were classified as animals with liver tumors. In other words, there are more situations in which animals will not develop liver tumors. Thus, female mice, which are exposed to multi-compounds at a dose level that indicated liver toxicity, and male mice will not develop a liver tumor in general.

In summary, the following concluding statement might be formulated: If multi-compounds cause liver tumors at all, then in female mice, which are exposed to a dose level that indicated liver toxicity in the dose finding study, and in male mice, which seem to be more sensitive in general (as also indicated in SET1). This observation may also support the intuitive argument that mice, which are exposed to any multi-compound, have a high chance to develop liver tumors as long as the dose level is high enough, i.e., as long as the liver weight significantly increases because the organ is overused.

### Comparison to Previous Work

To the best of our knowledge, there are only two previous approaches on the prediction of liver carcinogenicity. Allen et al. [20] considered results for mice from 83 NTP TRs and results for rats from 87 NTP TRs. Boobis et al. [21] considered results for mice and rats from 16 NTP TRs. However, both approaches employed prediction attributes based on summary statistics for entire 2Y-CSs, in contrast to the finer data level of individual animals in the present analysis.

In the work of Allen et al. [20], a significant increase in three histopathological findings (hepatocellular hypertrophy, hepatocellular cytomegaly, hepatocellular necrosis) in the dose finding study was employed to predict the decision on carcinogenicity after the 2Y-CS. This prediction approach achieved an accuracy of 81%, sensitivity of 63%, and specificity of 86%; the additional inclusion of a significant increase in liver weight achieved an accuracy of 69%, sensitivity of 95%, and specificity of 62%. (These numbers are not explicitly reported in the work of Allen et al. because studies on mice and rats were evaluated separately. For compatibility with the present analysis, they were computed by combining the results given in Table 3 (results for mice) and Table 4 (results for rats) in the work of Allen et al.) These results are similar to the present results for SET1. The best possible prediction accuracy was about 80%. Furthermore, there was also a considerable difference between sensitivity and specificity. Either many false positive decisions or many false negative decisions have to be accepted.

For comparisons, it should also be noted that the evaluation procedure was different. Allen et al. [20] selected the prediction attributes because the incidence of the three lesions (combined with increased liver weight) correlated with carcinogenicity in the considered TRs. Then, they predicted carcinogenicity using these attributes in the same TRs. In the field of statistical learning, it is known that this procedure may overestimate the true prediction performance [41, 44]. This is because the prediction attributes were selected with the knowledge that they correlate with carcinogenicity in the TRs in which the predictions will be performed. However, this does not simulate the application scenario in which carcinogenicity is unknown and should be assessed (and hence, the correlation is unknown). The present results, which were computed using a cross-validation, provide more realistic estimations of the predictive potential in real application scenarios.

In the work of Boobies et al. [21], a significant increase in two histopathological findings (hepatocellular hypertrophy and/or hepatocyte necrosis) combined with increased liver weight in the dose finding study was employed to predict the incidence of liver tumors. This approach correctly identified 12 of 13 substances (92% sensitivity) that caused liver tumors in at least one sex of one species. The authors noted that these results are similar to the work of Allen et al. [20]. They also concluded that the current endpoints in dose finding studies are not sufficient to identify all substances that have carcinogenic potential. However, in comparison to the present analysis, this work has also a more correlation-based character, since no separate training and testing sets were employed.

Regarding the structure of the decision trees, the present results are in accordance with previous results. For example, differences regarding the incidence of tumors in mice and rats are known, e.g., [45, 46]. This fact is reflected in the decision trees, which identified the species as the most informative attribute and selected it as the root node. Furthermore, differences regarding the incidence of tumors in females and males are also known, e.g., [46–48]. This fact is reflected in the decision trees, which identified the sex as the second most informative attribute (in the case of mice).

### Alternative Perspectives

The discussion so far showed that there is potential to predict liver tumors in 2Y-CSs. However, the predictive potential may also be interpreted from alternative perspectives.

First, the perspective that all prediction attributes are controlled by the conductor of the 2Y-CS. For example, consider SET2 in which sensitivity, specificity, and accuracy enable reasonable argumentation. The C4.5 decision tree suggested that the outcome for female mice in future 2Y-CSs on multi-compounds will depend on the dose level. However, the dose level is a variable factor in the design of a 2Y-CS. For example, the 2Y-CS on the Ginkgo multi-compound (TR No. 578) administered dose levels that indicated liver toxicity in all animal groups. In contrast, the 2Y-CS on the Ginseng multi-compound (TR No. 567) administered dose levels that did not indicate liver toxicity in any animal group. Thus, the dose level might be an unknowing factor that influences the outcome of the 2Y-CS.

Second, the perspective that there seems to be a bias regarding the incidence of liver tumors. This bias is expressed by the patterns that were extracted from the C4.5 decision trees. For example, male mice, which are exposed to any substance at a dose level that indicated liver toxicity, will likely develop a liver tumor. This bias should be considered in the statistical evaluation of a 2Y-CS. For example, a weighting factor might be introduced to account for this general tendency of male mice, or a higher significance level might be defined for male mice. Another bias, for example, is that rats, which are exposed to multi-compounds, will unlikely develop a liver tumor. This bias should also be considered in the statistical evaluation. For example, multi-compounds might be liver carcinogens even if there is only a small, non-significant increase in liver tumors in rats.

However, to the best of our knowledge, this bias is currently not considered in the decision process on carcinogenicity.

## Conclusion

The present study applied data mining methods to biobank data. It was shown that the incidence of liver tumors in 2Y-CSs can be predicted using findings from the preceding dose finding studies. This was particularly successful for 2Y-CSs on multi-compounds. Therefore, it may be speculated that the proposed approach can also be applied to similar settings, e.g., the examination of tumors in other organs.

However, the extracted patterns simultaneously indicated that there are situations in which liver tumors are likely to occurâ€š and situations in which liver tumors are unlikely to occur. These situations are independent of the actual subject of the 2Y-CS, namely the test substance for which carcinogenicity should be assessed. Hence, the incidence of liver tumors does not depend only on the test substance. Therefore, we recommend considering this bias if the hazard or risk of a substance is assessed on basis of a 2Y-CS.
